# The transcription factor MEF2A plays a key role in the differentiation/maturation of rat neural stem cells into neurons

**DOI:** 10.1016/j.bbrc.2018.04.125

**Published:** 2018-06-07

**Authors:** Bangfu Zhu, Ruth E. Carmichael, Luis Solabre Valois, Kevin A. Wilkinson, Jeremy M. Henley

**Affiliations:** School of Biochemistry, Centre for Synaptic Plasticity, University of Bristol, BS8 1TD, UK

**Keywords:** Neural stem cells, Neurons, Differentiation, Development, MEF2, Transcription factor

## Abstract

Neural stem cells (NSCs) are self-renewing multipotent stem cells that can be proliferated *in vitro* and differentiated into neuronal and/or glial lineages, making them an ideal model to study the processes involved in neuronal differentiation. Here we have used NSCs to investigate the role of the transcription factor MEF2A in neuronal differentiation and development *in vitro*. We show that although MEF2A is present in undifferentiated NSCs, following differentiation it is expressed at significantly higher levels in a subset of neuronal compared to non-neuronal cells. Furthermore, shRNA-mediated knockdown of MEF2A reduces the number of NSC-derived neurons compared to non-neuronal cells after differentiation. Together, these data indicate that MEF2A participates in neuronal differentiation/maturation from NSCs.

## Highlights

• Undifferentiated and differentiated neural stem cells (NSCs) express MEF2A *in vitro*.
• NSC-derived neurons express more MEF2A than NSC-derived glia.
• Ablating MEF2A reduces NSC differentiation into neurons.

## 1 Introduction

The highly conserved myocyte enhancer factor 2 (MEF2) family of proteins were first described as the transcription factors required for differentiation of myoblasts to myocytes in vertebrate muscle development [1]. It has subsequently been shown that they coordinate expression of a range of proteins in all eukaryotic cells [2] by controlling target gene transcription through the direct recruitment of a range of transcriptional co-activators [3] and co-repressors [4]. As a result, MEF2 proteins regulate a wide variety of cellular pathways including apoptosis and coordination of tissue-specific functions [5].

The four vertebrate MEF2 isoforms are classified MEF2A-D. Each isoform is comprised of highly conserved N-terminal MADS (MCM1-Agamous-Deficiens-SRF) and MEF2 domains that mediate the DNA binding and dimerization required for function, and a variable C-terminal transcriptional transactivation domain [6].

All MEF2 isoforms are extensively expressed in the brain [7]. Interestingly, however, defined expression patterns during pre- and postnatal development suggest each isoform may have distinct roles at different stages of neuronal maturation. For example, while MEF2C expression remains relatively constant, MEF2A and D expression increases as neurons differentiate and mature [8]. Genome-wide analysis and functional characterization of cultured neurons has identified multiple MEF2-regulated genes associated with neuronal and synaptic development [9,10]. MEF2A in particular is highly expressed in neurons during synaptogenesis and plays important roles in the control of pre- and postsynapse formation [[9], [10], [11], [12], [13]]. Inducible *Mef2a, -c*, and *-d* knockout mice for temporal and cell-specific MEF2 deletion have been used to monitor the progression of hippocampal adult neural stem/progenitor cells, and have shown that each knockout can generate mature neurons. However, these neurons exhibit stunted dendrites, suggesting possible roles for MEF2 proteins in the control of adult hippocampal neurogenesis and dendritogenesis *in vivo* [14].

In this study, we focused on the roles of MEF2A in defining the differentiation, maturation and fate of NSCs in culture. We show that both undifferentiated and differentiated NSCs express MEF2A, with a subset of neuronally differentiated cells expressing significantly more MEF2A than their non-neuronal counterparts. Moreover, we show that specific ablation of MEF2A reduces the proportion of NSCs that differentiate into neurons.

## 2 Methods

### 2.1 Molecular biology

Cloning of all constructs was carried out using standard molecular biology methods. An shRNA sequence targeting rat MEF2A (GGGCAGTTATCTCAGGGTTCAA) or a non-targeting control (AATTCTCCGAACGTGTCAC) under the control of a H1 promoter were cloned into a modified form of the pXLG3 vector [15,16] expressing an EGFP marker.

### 2.2 Cell culture

NSCs were isolated from E14 Wistar rat embryos. Forebrain areas were dissected and dissociated with StemPro Accutase solution (Gibco), before being seeded into culture flasks. Cells were initially grown as neurospheres in proliferation media (DMEM/F-12 media + B27 supplement (Gibco), 1 × Insulin-Transferrin-Sodium Selenite Supplement (Gibco), 1 × Non-Essential Amino Acids Solution (Gibco), 20 ng/ml of each EGF/bFGF (Preprotech), 1 × penicillin/streptomycin (Sigma Aldrich)).

For NSC differentiation, the media was replaced with differentiation media (proliferation media lacking EGF and bFGF and supplemented with 200 μM Ascorbic acid (Sigma Aldrich)) and the cells were plated on PDL-coated glass coverslips.

All neural cell transfections were performed using Lipofectamine 2000 (Invitrogen) according to the manufacturer's instructions.

### 2.3 Immunocytochemistry and immunofluorescence imaging

Immunocytochemistry assays were performed with paraformaldehyde fixation according to standard protocols [17]. Briefly, cells were fixed using 4% PFA before or after differentiation and permeabilised using Triton ×100 (0.1%). 10% horse serum in PBS was used to block cells and antibody staining was performed with the following antibodies: Nestin (rabbit polyclonal, Sigma-Aldrich SAB4200394, 1:500), Tuj1 (mouse monoclonal, Sigma-Aldrich T8578, 1:200), GFAP (rabbit monoclonal, Cell Signaling Technologies 12389, 1:200), MEF2A (rabbit monoclonal, Abcam ab109420, 1:100). The secondary antibodies were Cy3 donkey anti-rat IgG and/or Cy2 donkey anti-mouse IgG (Molecular Probe, Invitrogen). The samples then were mounted in Fluoromount-G mounting medium (eBioscience Inc.). Nuclei were counterstained with diamidino-2-phenylindole (DAPI).

All immunofluorescence images were taken using a Leica SP5-II confocal laser scanning microscope attached to a Leica DMI 6000 inverted epifluorescence microscope (Wolfson Bioimaging Facility, University of Bristol), and processed and analysed using ImageJ software.

### 2.4 Data analysis

All quantified data are presented as the mean ± SEM from at least three independent cultures, and where necessary results were normalised to the average of the control values (set to 100%) for each batch of cells. Statistical analysis was performed using Graphpad Prism software (Graphpad Inc.). Statistical significance was placed at a p value of less than 0.05.

## 3 Results and discussion

### 3.1 MEF2A is expressed in undifferentiated and differentiated NSCs

**Fig. 1:**
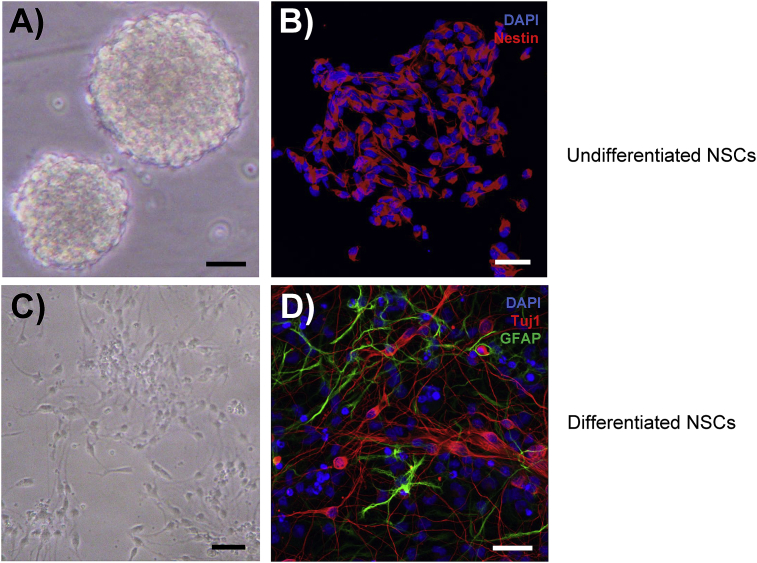
NSC culture and differentiation. A). NSCs were isolated from rat embryonic brains and grown as neurospheres. B). NSCs were identified by immunostaining with the neural stem cell marker Nestin (red). Nuclei were stained with DAPI (blue). C). Following withdrawal of growth factors, NSCs are induced to differentiate. D). Immunostaining showing differentiation of NSCs into neurons (Tuj1 staining in red) and glia (e.g. astrocytes in green with GFAP staining).

MEF2A is highly expressed in the brain where it plays key roles in neuronal maturation, dendritic morphogenesis and synapse formation [5,8,9,12]. However, it has not been reported if MEF2A is expressed in NSCs *in vitro*. To answer this question, we examined the expression of MEF2A in both undifferentiated and differentiated NSCs by immunocytochemistry. Rat NSCs isolated from E14 embryonic forebrains and cultured in DMEM/F12 medium supplemented with growth factors (bFGF2 and EGF) proliferate rapidly to form neurospheres (Fig. 1A–B) [18]. On growth factor withdrawal they then differentiate into neurons and glial cells (Fig. 1C–D).

**Fig. 2:**
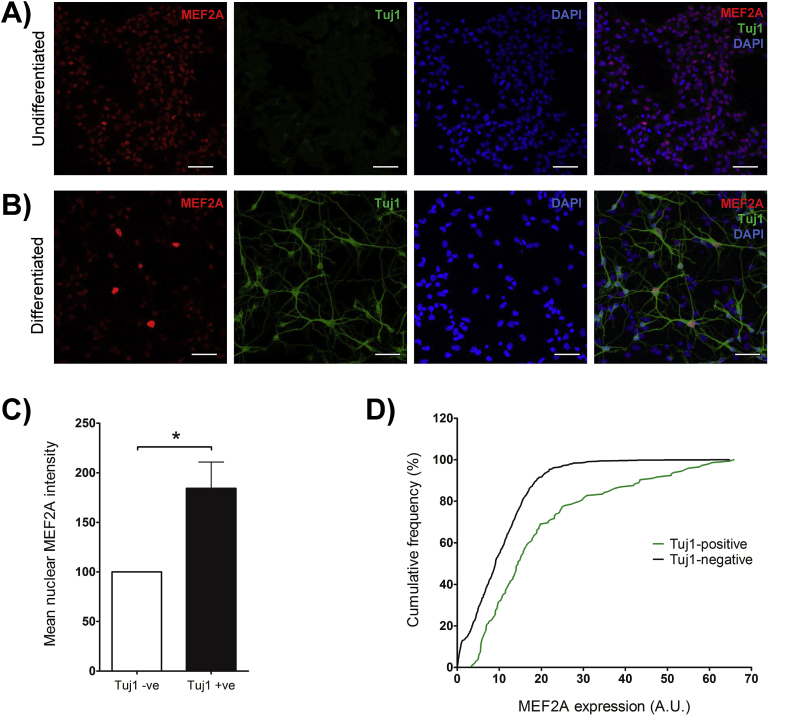
MEF2A expression in NSCs. A). MEF2A (red) is present in undifferentiated NSCs, although the relative levels of expression appear variable. B). MEF2A (red) is expressed at a significantly higher level in Tuj1-positive neurons (green), as opposed to surrounding Tuj1-negative cells, in differentiated cultures. Nuclear staining is shown with DAPI (blue). C). Quantification of MEF2A expression in Tuj1-positive and -negative cells following differentiation. n = 5 independent cultures analysing 20–40 Tuj1-positive and 50–100 Tuj1-negative cells from each, one-sample *t*-test p = 0.034. D). Cumulative frequency of MEF2A expression in differentiated Tuj1-negative (black line) and Tuj1-positive (green line) cells. The data quantify MEF2A expression in 670 Tuj1-negative and 145 Tuj1-positive cells from 5 independent cultures.

We stained for MEF2A in undifferentiated NSCs and after growth factor withdrawal–evoked differentiation (Fig. 2). Although MEF2A is expressed in the nuclei of undifferentiated NSCs, the expression level appears to be relatively heterogeneous within the population (Fig. 2A). MEF2A is also present in differentiated cells after 4 days of growth factor withdrawal and the mean expression level is significantly higher in the nuclei of Tuj1-positive neuronally differentiated cells, compared to neighbouring Tuj1-negative cells (Fig. 2B–C), suggesting that, as a population, MEF2A expression is increased in neuronal compared to non-neuronal cells.

However, since MEF2A expression was not increased in all Tuj1-positive cells compared to nearby Tuj1-negative cells, we wondered whether differentiation into neurons increased the proportion of cells expressing comparatively high levels of MEF2A. To examine this directly, we analysed MEF2A expression in individual Tuj1-positive and Tuj1-negative differentiated cells and present our data as a cumulative frequency graph (Fig. 2D). Our data show that although some Tuj1-positive differentiated cells express low levels of MEF2A, the shift to the right on the cumulative frequency plot demonstrates that a much greater proportion of differentiated Tuj1-positive neuronal cells express higher levels of MEF2A compared to differentiated Tuj1-negative cells. Together, these imaging data indicate that expression of a differentiated neuronal marker correlates with higher levels of nuclear MEF2A, particularly within a subpopulation of neuronally differentiated cells. This suggests that MEF2A could play a role in the formation, maintenance or function of NSC-derived neurons.

### 3.2 MEF2A promotes NSC differentiation/maturation into neurons

**Fig. 3:**
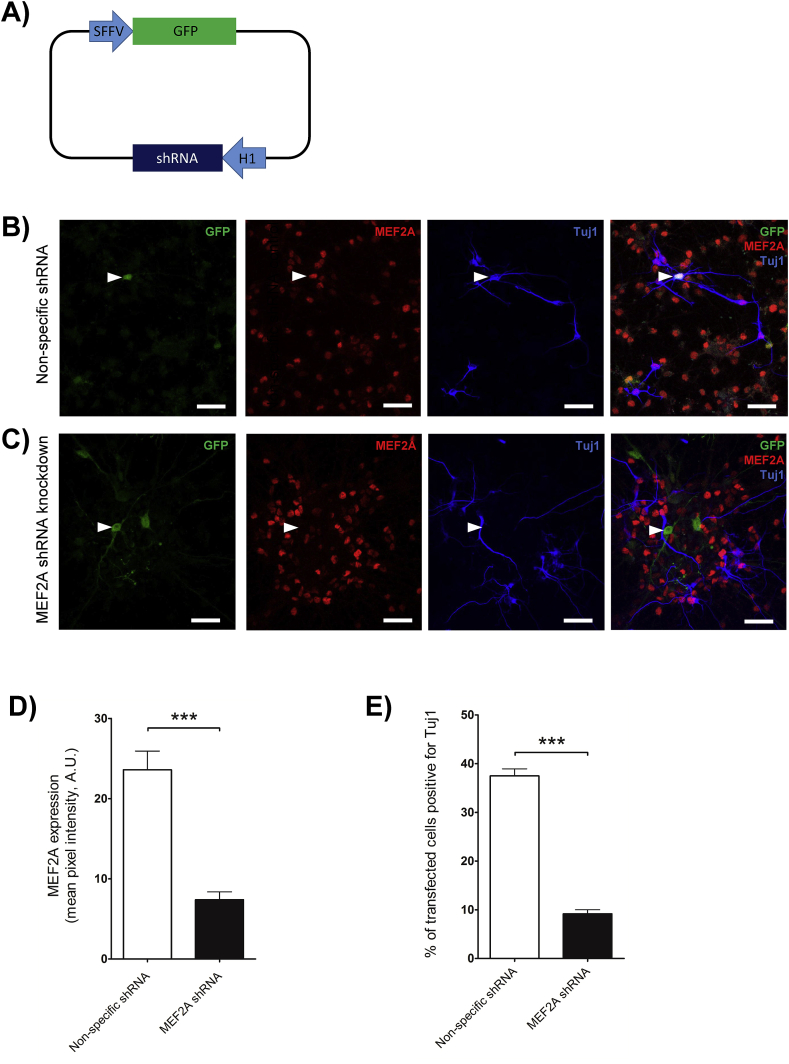
MEF2A knockdown reduces NSC differentiation into neurons. A). Map of the transfection vector, expressing an shRNA (either a MEF2A-specific shRNA or a non-specific control) under a H1 promoter as well as GFP under a SFFV promoter as a reporter of transfection. B & C). NSCs transfected with non-specific shRNA (B) or MEF2A-specific shRNA (C) prior to differentiation, stained for MEF2A (red) and Tuj1 (blue). Example transfected cells (GFP-positive) are highlighted by white arrowheads. D). MEF2A expression in NSCs transfected with MEF2A shRNA was almost completely ablated compared to NSCs transfected with a non-specific shRNA (n = 5 independent cultures analysing 14 transfected cells per condition in total, Student's unpaired *t*-test p = 0.0002). E). Fewer MEF2A shRNA transfected cells differentiated into Tuj1-positive neurons compared to NSCs transfected with a non-specific shRNA control (n = 3 independent cultures analysing 30–40 transfected cells per condition from each, Student's unpaired *t*-test p < 0.0001).

To define if MEF2A is involved in driving NSCs towards a neuronal lineage, we next transfected undifferentiated NSCs 24 h after plating with a vector encoding both an shRNA to knock down expression of endogenous MEF2A, and GFP to identify transfected cells (Fig. 3A).

Although transfection levels are relatively low, typically ∼5% of NSCs, this is an advantage for imaging studies because it allows direct comparison of transfected (GFP-expressing, green) and non-transfected (non-green) NSCs on the same coverslip.

Untransfected cells show strong endogenous MEF2A staining, as seen previously (Fig. 3B and C). However, MEF2A staining was not detectable in any cells transfected with the shRNA targeting MEF2A (Fig. 3C, quantified in Fig. 3D), consistent with a recent report showing that this MEF2A shRNA construct is highly effective at knocking down MEF2A expression in rat neurons by Western blotting and immunofluorescence [19]. In contrast, cells transfected with control non-specific shRNA show strong endogenous MEF2A immunostaining comparable to levels in untransfected cells (Fig. 3B).

Quantitative analysis of these data demonstrate that following growth factor withdrawal for 4 days, only 9.2 ± 0.8% of NSCs transfected with the MEF2A shRNA developed into Tuj1-positive cells compared to 37.5 ± 1.4% of NSCs transfected with the non-specific shRNA control (Fig. 3E), indicating that MEF2A promotes a neuronal phenotype following NSC differentiation.

Since rat NSCs can differentiate into neurons or glia *in vitro*, they are a useful model to investigate neuronal differentiation and development. Consistent with a report that MEF2A expression increases as cerebellar granule neurons differentiate and mature, which is marked by neurite growth [8], we show that MEF2A is present in NSCs and differentiated cultures, with significantly elevated expression in differentiated neurons. In addition, we demonstrate that shRNA knockdown of MEF2A decreases NSC differentiation to neural stem/progenitor cell-derived neurons, supporting the notion that MEF2A drives NSC differentiation into neurons *in vitro*.

We propose that the transcriptional activity of MEF2A makes it a likely regulator of the changes in gene expression that underpin neuronal differentiation of NSCs. However, our present data do not rule out the alternative possibility that MEF2A could be important for the development or survival of Tuj1-positive neurons after differentiation. Indeed, it has previously been reported that inhibition of MEF2 in cultured cortical neurons leads to apoptotic cell death, suggesting that MEF2 is necessary for neuronal survival, particularly of newly-differentiated neurons [20,21].

The mechanisms of how MEF2A might regulate the differentiation/survival of neurons during brain development, and how it is itself regulated, are the subject of intensive research. In synapse development, for example, the transcriptional activity of MEF2A may be modulated by posttranslational modifications such as phosphorylation and/or SUMOylation [9,10]. Further research is needed to resolve how MEF2A is controlled on a temporal and cell-type specific level to regulate its roles in development.

Nonetheless, our data are the first demonstration that MEF2A is expressed in embryonic brain-derived NSCs cultured *in vitro* and plays a role in their differentiation into a neuronal phenotype. Thus, we confirm and extend previous studies demonstrating the importance of MEF2A as an arbiter of neuronal development and maturation. These findings are of potential importance because NSCs can be rapidly grown and easily manipulated so deeper understanding of the factors driving NSC differentiation could open new avenues to develop novel cell replacement therapies.

## Funding

We are grateful to the BBSRC and Parkinson's UK (G-1605) for supporting this research. Imaging was carried out at the Wolfson Bioimaging Facility, University of Bristol, UK.
